# Commentary on ‘corradjust unveils biologically relevant transcriptomic correlations by efficiently eliminating hidden confounders’

**DOI:** 10.1093/nar/gkag932

**Published:** 2026-10-05

**Authors:** Anahita Babaie, Enakshi Saha, John Quackenbush, Marouen Ben Guebila

**Affiliations:** Department of Mathematics, Brandeis University, Waltham, MA 02453, USA; Department of Epidemiology and Biostatistics, University of South Carolina, Columbia, SC 29208, USA; Department of Biostatistics, Harvard T.H. Chan School of Public Health, Boston, MA 02115, USA; Department of Data Science, Dana-Farber Cancer Institute, Boston, MA 02215, USA; Channing Division of Network Medicine, Brigham and Women’s Hospital, Boston, MA 02115, USA; Department of Biostatistics, Harvard T.H. Chan School of Public Health, Boston, MA 02115, USA; Department of Medical Oncology, Dana-Farber Cancer Institute, Boston, MA 02215, USA

## Introduction

Modeling covariates and correcting systematic variation in biological networks derived from high-dimensional gene expression data is essential for the discovery of meaningful biological insights during subsequent analyses. There has been extensive work in batch correction with genomic data, such as RNA expression, where there are well-established principles that are now standard elements of any robust analytical pipeline. When confounding variables are known, regression-based methods [1, 2] can be used to estimate their effects and derive a correction. However, in cases where these covariates are unknown, other methods have been developed that “protect” the known biological signal by deriving residuals that control for the effects of confounding variables [3].

Batch correction for networks has been less fully explored but generally builds on applying the same principles to higher-order measurements, such as correlation networks of gene expression. We previously developed COBRA (co-expression batch reduction adjustment) as a method that explicitly models covariates [4] in co-expression networks. COBRA takes a gene expression matrix and a design matrix of known sample covariates (such as batch, sex, or age) as inputs, computes the overall sample co-expression matrix and performs an eigen decomposition on it, retaining only the eigenvectors corresponding to nonzero eigenvalues—a step that reduces the parameter space from the full quadratic number of gene pairs down to a much more manageable number proportional to the sample count. It then estimates a set of pseudo “eigenvalues” for each covariate by solving a least-squares regression problem that minimizes the error in reconstructing the co-expression matrix as a weighted linear combination of covariate-specific components. This decomposition yields one co-expression submatrix per covariate in the design matrix, where each submatrix captures the portion of gene-gene correlation structure attributable to that variable. To produce a batch-corrected co-expression matrix, the components corresponding to batch (and any other unwanted covariates) are simply subtracted out, leaving behind only the biologically relevant correlation structure—for example, the component associated with a disease/control comparison. In practice, we find that analyses using COBRA to do batch correction as part of the workflow yield enrichment of fewer significant gene sets—but gene sets that are of high biological relevance that inform our understanding of the systems under study.

Recently, Nersisyan *et al*. described CorrAdjust [5], a method for batch correction in correlation networks that does not require prior knowledge of covariates. CorrAdjust addresses putative batch effects by prioritizing protection of associations within previously defined biological process or pathway categories. CorrAdjust takes as input a gene expression matrix and a reference collection of pathway- or process-gene pairs, then ranks all gene pairs by their Pearson’s correlation and measures how well the known reference pairs are concentrated at the top of that ranking using a per-gene hypergeometric enrichment score. It uses this score as an optimization target in a greedy search, iteratively selecting whichever principal component of the expression data, when regressed out, most improves the enrichment of reference pairs among the top-ranked correlations. This process repeats—removing one PC per iteration from a possibly nonconsecutive set—until the score plateaus, at which point the corrected expression matrix (with those confounding principal components, or PCs, removed) reflects biologically meaningful correlations rather than technical or hidden biological artifacts. To ensure the results generalize, both gene pairs and samples are split into training and validation/test sets, and a permutation-based negative control confirms that the enrichment signal is real rather than an artifact of the optimization. This introduces a bias that promotes gene pairs that are annotated to co-occur in known pathways or biological processes. We will refer to both canonical pathway and biological process annotation as “pathways.”

In assessing the performance of CorrAdjust, the developers demonstrated that its use yielded high enrichment scores for large numbers of pathways (a high GES score per tissue) but did not examine whether the highly ranked pathways were relevant in the biological context of the analysis being performed. This missing qualitative assessment reflects a “quantity over quality” approach that may lead to statistically significant pathways but does not qualify the biological relevance of “significant” processes.

We compared CorrAdjust and COBRA correlations in removing systematic biases in the data and recovering meaningful correlations. For both, we ran them with parameters used by Nersisyan et *al*. (samples, genes, pathways) for their analysis, including the use of sex, age, RNA integrity number (RIN), and postmortem interval (PMI) as covariates for COBRA.

We used the analytical pipeline described by Nersisyan *et al*., starting with gene expression data from which we calculated correlation matrices, applied either CorrAdjust or COBRA, and performed both preranked gene set enrichment analysis (GSEA; [6]) using the enrichment score for each gene across all tissues and over-representation analysis (ORA; [7]) using the 100 most significant genes; for both, we used the MSigDB canonical pathways (CP) annotation. We also did the same analysis using GO biological process annotation. As a note, both these pathway annotations were used to optimize CorrAdjust’s internal procedure. As a check on our reimplementation of the full pipeline used by Nersisyan *et al*., we compared our results to Fig. 5 in their original paper (Fig. 1). In using CorrAdjust, we found we were only able to reproduce their results for data from GTEx (we had to override the automatic PC selection for thyroid to recreate the four PCs that they reported, as suggested by them). In our hands, we could not reproduce the results for data from TCGA and the Geuvadis consortium, possibly because of their use of pipelines for data processing and normalization for small RNA counts that were not disclosed in their paper. Consequently, we only report results for the 11 GTEx tissues.

**Figure 1.**
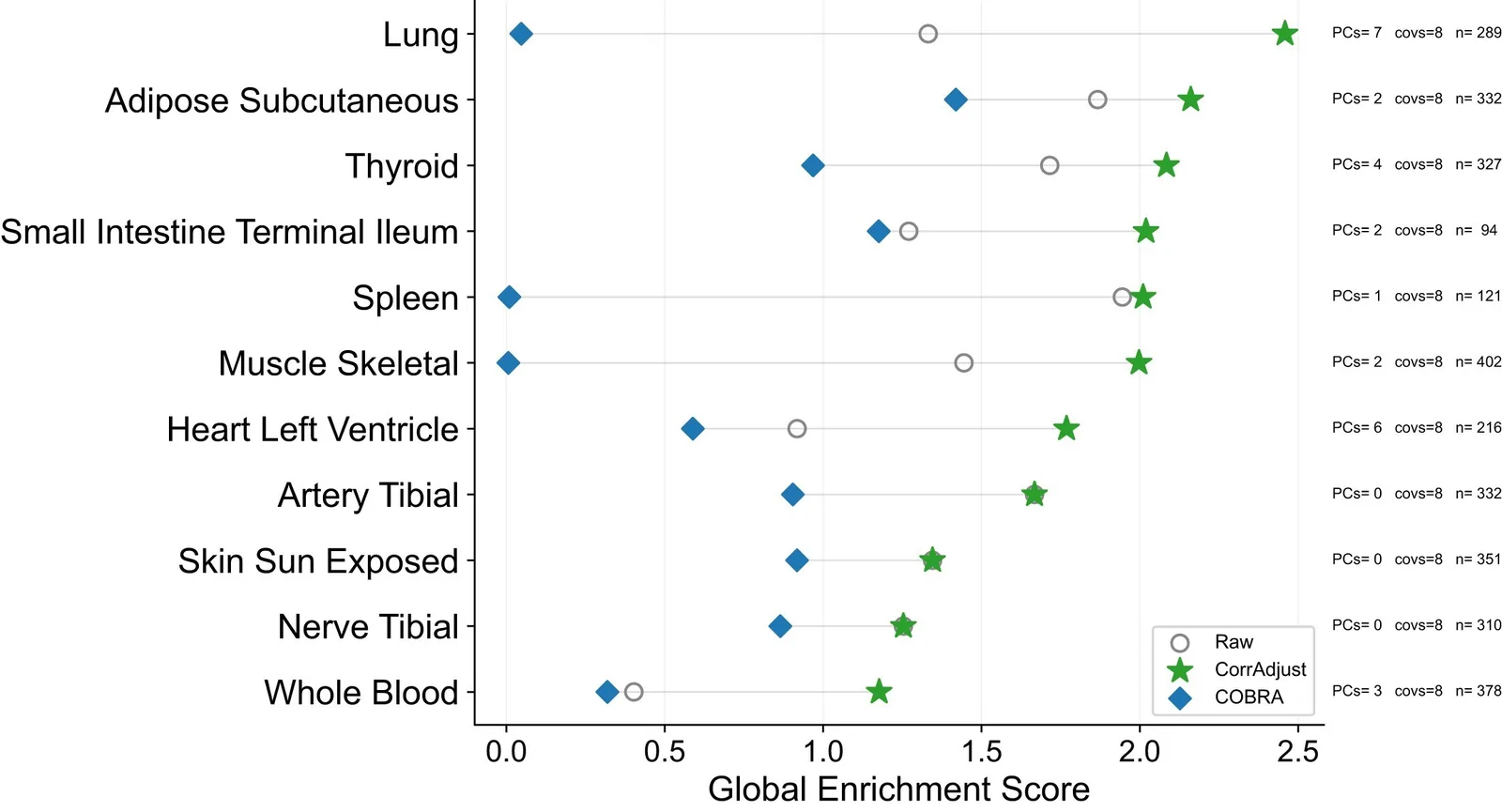
We used COBRA and CorrAdjust to reproduce the bottom panel of fig. 5B of Nersisyan *et al*. for these two methods applied to remove batch effects for eleven tissues in GTEx. Global enrichment scores are shown for the raw correlation matrix and those following CorrAdjust and COBRA corrections. Although COBRA finds fewer significant pathways to be enriched, reflecting a lower Global Enrichment Score, the pathways are more specific to the known biological processes active in the respective tissue.

For both CorrAdjust and COBRA, and in ORA (Supplementary Fig. S1) and GSEA (Supplementary Fig. S2) analyses, we identified common and nonspecific terms that were shared between tissues—a result that is not unexpected given the high level of correlation in expression of housekeeping and other genes associated with basic cellular processes. The one notable exception in which tissue-specific functional groups were identified was the appearance of heme-related terms in GTEx whole blood (Supplementary Fig. S1).

We have encountered this in other situations and have suggested using modules (communities or subnetworks of highly connected network elements) to identify biological processes that may be coordinately regulated [8, 9]. Our typical pipeline first takes in a biological network, runs a community detection algorithm, and then performs ORA on the genes in each module. Our approach is based on the assumption that physiological functions in each tissue are carried by specialized and distinct functional modules [10]; this is the process we used in our COBRA paper that allowed us to find both thyroid tissue-specific and cancer-specific terms in thyroid cancer [4].

Using the authors’ chosen parameters for network reconstruction, we identified modules in COBRA and CorrAdjust networks using WGCNA to detect modules of correlated genes after which each module was assessed for overrepresentation of genes in specific pathways. All tissues but COBRA-spleen identified rich modules across the same number of genes (Fig. 2). Our hypothesis is that accurate network inference methods should produce nonredundant, precise, and specific genetic submodules, each carrying a biological function, with at least one module representing a primary tissue-specific function such as fatty acid metabolism in adipose tissue or insulin production in pancreas. To assess the meaningfulness of the identified modules, we then ran ORA on the modules using gene ontology-biological process (GO-BP) annotation (Supplementary Fig. S3). As we expected, the modules found using both COBRA and CorrAdjust are enriched for genes associated with tissue-specific processes. So both methods find meaningful signals. The question is whether maximizing for concordance with GO or CP pathways enhances noise by producing modules containing genes representing processes that are either nonspecific or not relevant to the system being analyzed.

**Figure 2.**
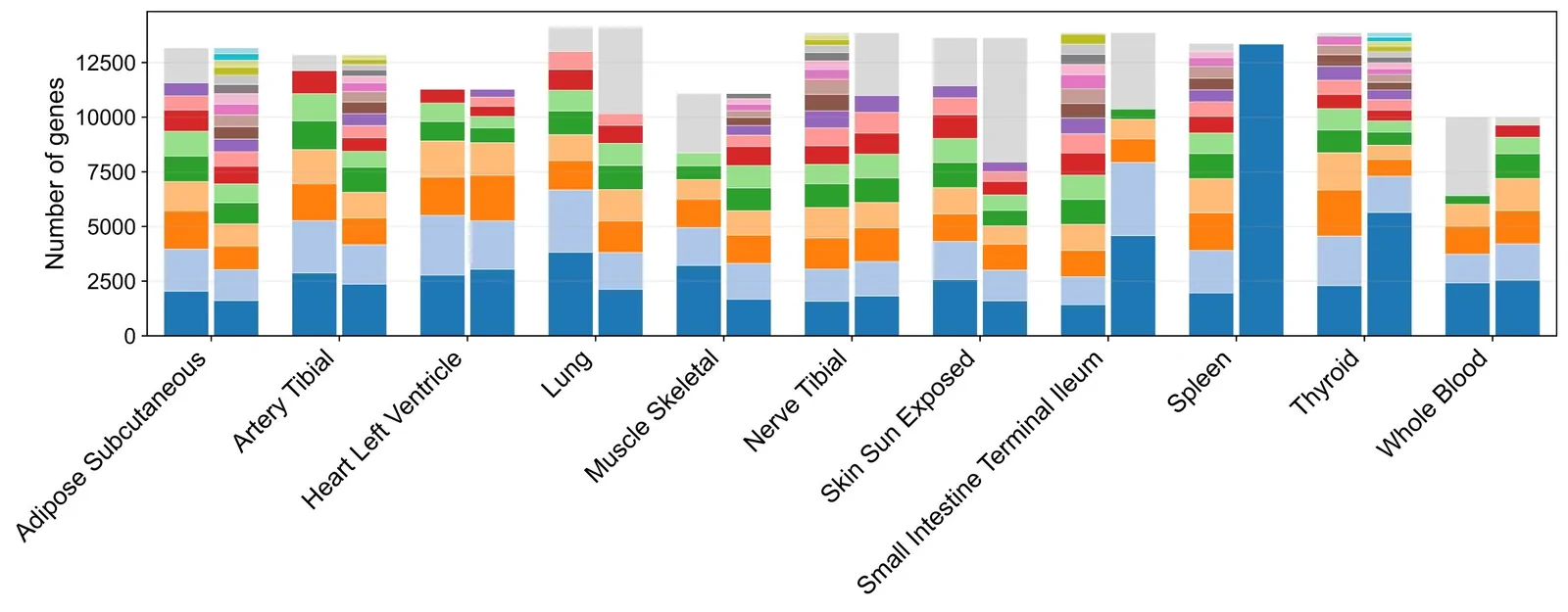
Module sizes in CorrAdjust (left) and COBRA (right). Each tissue has two bars, representing modules in CorrAdjust (left) and COBRA (right). The stacked bars have the same length because both networks have the same number of genes. Each network has a different number and size of modules, represented by different colors. Grey sets are genes unassigned to any module.

Although we used manual curation in creating Supplementary Fig. S3 (an approach we have used in other published studies [8, 9], here, we chose to use a principled, automated approach. Using a set of tissue-specific keywords (like digestion for intestine and epiderm for skin, Supplementary Table S1), for each module we computed the “precision” of the functional enrichment defined as the number of terms corresponding to tissue-specific functions and divided that by the entire collection of overrepresented terms. To benchmark other contexts, such as cancer tissues, similar ground truth annotations can be defined. We found that for 6 of 11 GTEx tissues, COBRA-derived modules are more precise for tissue function (using best module score) (Fig. 3A), such that they are enriched for biological terms specific to the tissue being analyzed, without significant numbers of additional nonspecific terms in that module. We chose best module score as a reflection of our model of highly specialized functional modules specific to each tissue. This means that the COBRA modules are composed of correlated sets of functionally enriched genes. It should be noted that CorrAdjust performed almost as well, outperforming COBRA in 5 of the 11 tissues.

**Figure 3.**
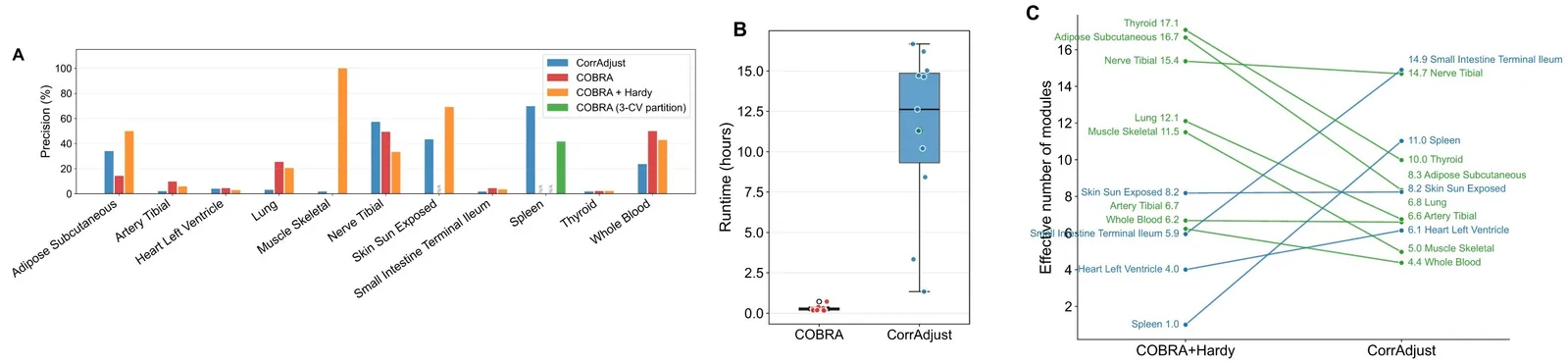
Comparative analysis of precision and performance of COBRA and CorrAdjust networks. **(A)** Precision of identified tissue-specific modules across 11 GTEx tissues in COBRA versus CorrAdjust in the best scoring module. Solid red represents COBRA scores on the original covariates; orange bars additionally include Hardy scores; green bar report three-fold cross-validation performance. **(B)** Runtime benchmark shows more >20× speedup for COBRA in comparison to CorrAdjust. **(C)** Number of effective modules in COBRA and CorrAdjust networks.

We noticed, however, that many of the terms we were finding were related to possible degradation of postmortem samples. We found that if we added an additional covariate to the analysis, Hardy scale (which quantifies the quality of postmortem tissue) as a covariate in COBRA makes modules even more precise for 2 additional tissues (now 8 of 11 COBRA tissues have the highest precision). We expect that the inclusion of additional standard covariates such as race, ethnicity, and batch would yield even more meaningful results.

It is worth noting that COBRA did not perform as well as CorrAdjust for spleen, which is one of the GTEx tissues with the fewest number of samples (241) of which only 50% (121) were used for training. This is not surprising; however, given that COBRA is a regression method, and so is sensitive to sample size. A COBRA run with three-fold cross validation (161 training samples) produced valid module partitions (Fig. 3A).

We ran all these analyses using the same parameters chosen by Nersisyan et *al*. (samples, tissues, genes, pathway annotation) so that none of our choices favor COBRA. We note that several of these choices, such as the gene filter (median TPM of 1, ∼10k gene), keep CorrAdjust runtime tractable, and the 50% sample partition exists because it requires cross validation. COBRA, a closed-form covariate regression, requires neither and works at genome scale for all samples. Indeed, in addition to resolving spleen modules with a few more samples, increasing the number of input genes from the ∼10k selected by Nersisyan et *al*. to almost 20k genes (with a median count of 1), COBRA converges in less <20 min per tissue, while CorrAdjust averages 14 h per tissue, requiring a full week for the collection of eleven tissues to be analysed (Fig. 3B). We also computed the number of effective modules as the exp(H), with H the Shannon entropy across genes, and found that COBRA had more balanced module distribution in 7/11 tissues (Fig. 3C).

In comparing CorrAdjust to COBRA, Nersisyan *et al*. questioned whether the batch correction learned on one “training set” could be transferred to a second “test set.” We show that test eigenvalues are predictable using COBRA parameters learned in a training set. We do this for the default maximum likelihood estimation (MLE) method but also extend to two new modes. Part of the reason for this is the fact that the covariate-derived MLE networks in COBRA do not always produce positive eigenvalues, an essential condition for reconstruction of positive semidefinite matrices (such as correlation matrices). To address this concern, we added two additional options for estimating and subtracting correlations depending on covariates such as batch: nonnegative least squares (NNLS), and nonnegative L1-regularized coefficients (NNLASSO), both available in netZooPy v0.11 [11]. We note that these two modes were not used in any of the direct comparisons presented here, which rely on the published COBRA estimator; we introduce them solely to address the point raised by Nersisyan *et al*. regarding generalization of COBRA. To assess the performance of MLE, NNLS, and NNLASSO regression, we divided the data for each GTEx tissue into training and test sets (using the same sample partitions defined by Nersisyan et *al*.). We fit a COBRA model with the four covariates used by Nersisyan *et al*. for their benchmark to the training set and applied it to the test set using the COBRA trained model to predict the co-expression matrix using test set data and measured Pearson’s *r* between predicted values and measured values in the test co-expression matrix across 11 tissues as a way of assessing accuracy. That is, we assess whether the covariate model learned on the training samples generalizes to unseen samples by predicting their co-expression matrix. As can be seen in Fig. 4, MLE, NNLS, and NNLASSO produce similar results in predicting the test co-expression matrix, and they all had a good performance because of the strong linearities in gene expression data, further highlighting that MLE mostly picks positive coefficients (a fact we demonstrated in Table S1 in Micheletti et *al*.). What is important here, however, is that all three implementations performed well in transferring training results to a related test set, indicating that they did a good job of estimating the source of covariate-driven correlation in expression data. MLE reconstructs the original co-expression matrix exactly because it is unconstrained in the type of matrix it produces thereby producing indefinite estimates as opposed to NNLS and NNLASSO that do constrained optimization which produces valid covariance matrices.

**Figure 4.**
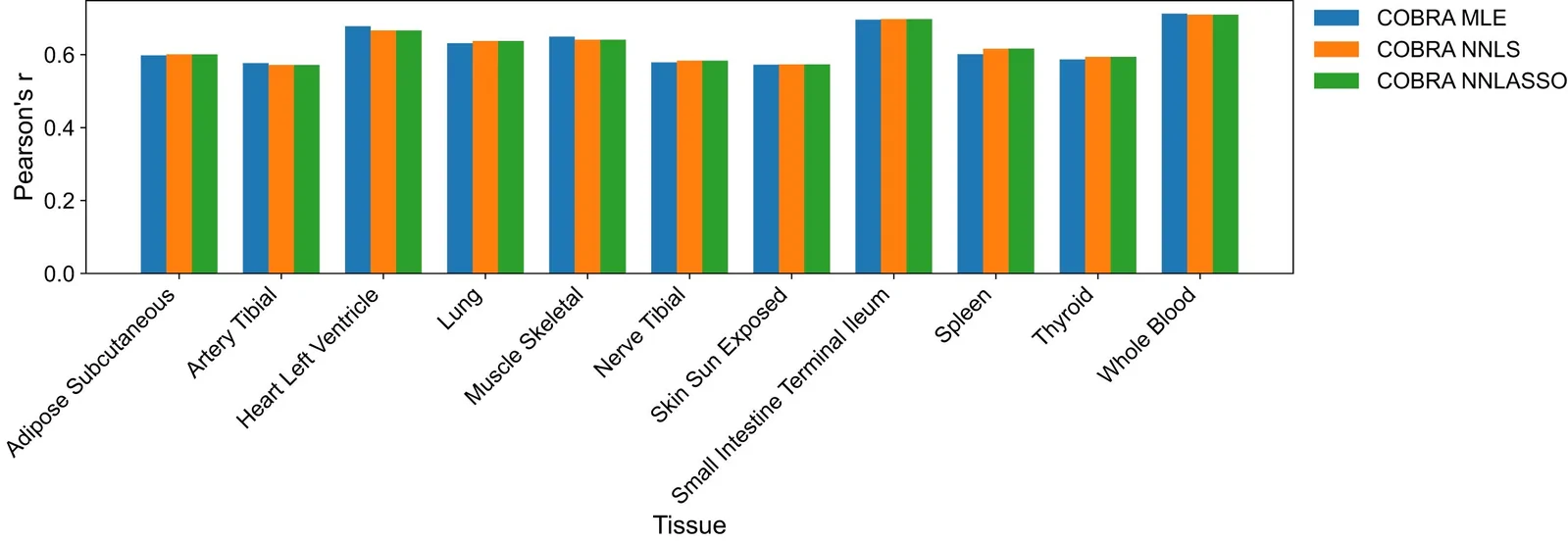
Performance of learned COBRA models (MLE, NNLS, NNLASSO) in the test data set. The bar plots represent Pearson’s *r* values for the prediction of covariance matrices in the test data set (using partitions defined in Nersisyan *et al*.) [5] using COBRA parameters from the training data set in 11 GTEx tissues. Pearson’s *r* is computed between the observed test set covariance matrix and the covariance matrix predicted from the training set.

Performance issues aside, we believe that COBRA’s explicit definition of covariates has conceptual advantages over CorrAdjust’s maximization of pathway database representation. In some sense the use of a “liver-specific pathway” in tissues other than liver introduces a different sort of confounding because it collects a group of genes in a way that may not be representative of processes in that tissue and maximizes concordance within that pathway or set of genes that are not necessarily relevant to processes in that tissue or biological state.

## Conclusions

Although it has long been known that gene expression can be affected by covariates and manifest as batch effects, it is only recently that we have come to recognize that there are higher order effects of batch and other covariates on correlation structure that must be corrected for. CorrAdjust presents a new interesting angle to tackle this issue based on preserving signal in known pathways but does not provide estimates of signal and noise in tissue contexts. COBRA, in contrast, uses a simple, regression-based model to identify and adjust for unwanted effects of covariates such as batch in a way that can lead to discovery of meaningful correlations by emphasizing unbiased reduction of noise. Our argument here is based on the quality of discoveries rather than quantity.

## Supplementary Material

gkag932_Supplemental_Files

## Data Availability

Code and data necessary to reproduce the analyses we present here are available in Zenodo (https://doi.org/10.5281/zenodo.20187236). This paper is linked to: https://doi.org/10.1093/nar/gkag931.
