# Supplementary material for: Commentary on ‘corradjust unveils biologically relevant transcriptomic correlations by efficiently eliminating hidden confounders’
Source: Nucleic Acids Res. 2026 Oct 5;54(18):gkag932. doi: 10.1093/nar/gkag932 (PMC13635743; doi:10.1093/nar/gkag932)
Supplement: Supplementary file 1 [file gkag932_supplemental_files.zip › figS1.pdf]

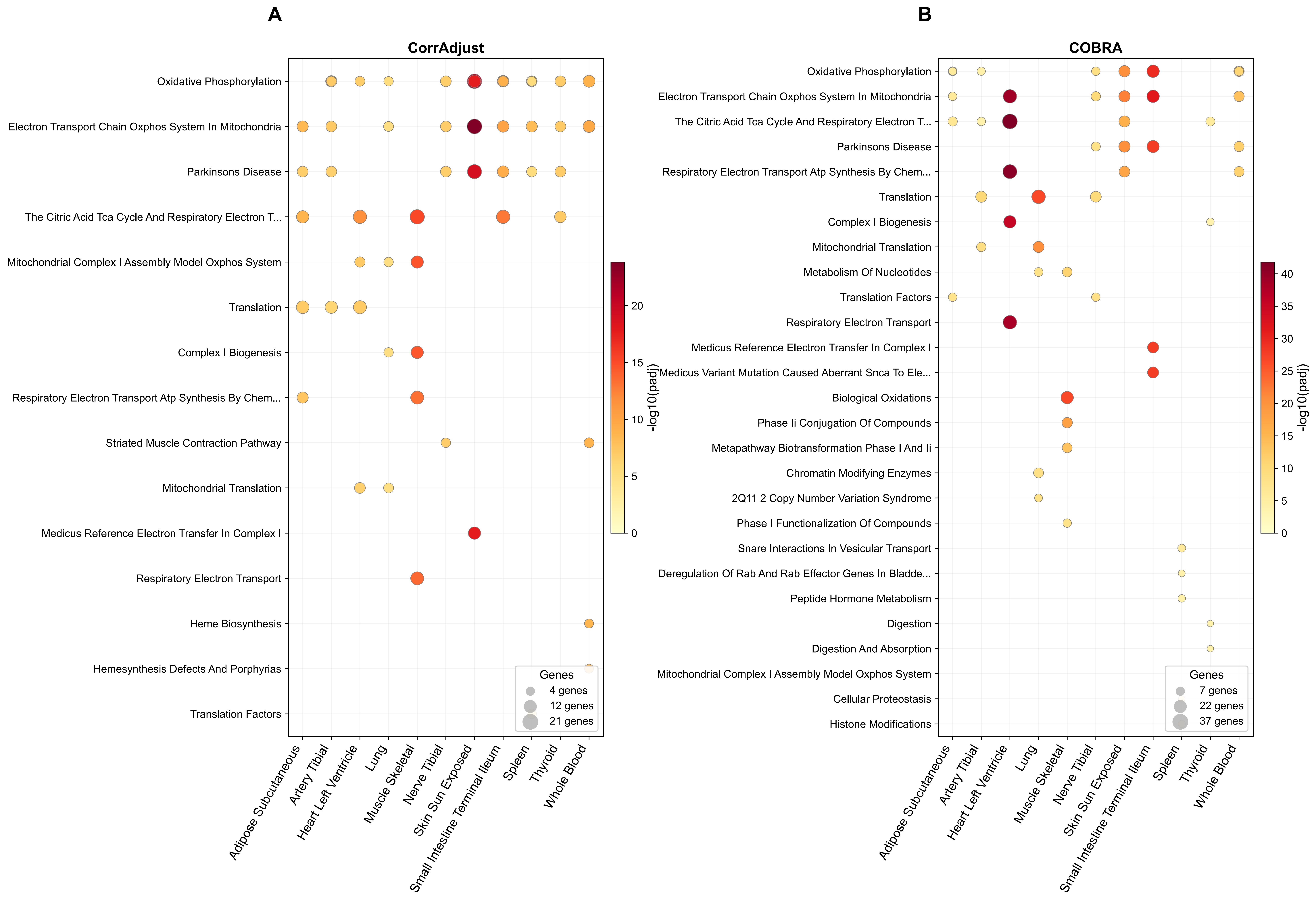
A
CorrAdjust
Oxidative Phosphorylation
Electron Transport Chain Oxphos System In Mitochondria
Parkinsons Disease
The Citric Acid Tca Cycle And Respiratory Electron T...
Mitochondrial Complex I Assembly Model Oxphos System
Translation
Complex I Biogenesis
Respiratory Electron Transport Atp Synthesis By Chem...
Striated Muscle Contraction Pathway
Mitochondrial Translation
Medicus Reference Electron Transfer In Complex I
Respiratory Electron Transport
Heme Biosynthesis
Hemesynthesis Defects And Porphyrias
Translation Factors
Genes
4 genes
12 genes
21 genes
-log10(padj)
0
5
10
15
20
Adipose Subcutaneous
Artery Tibial
Heart Left Ventricle
Lung
Muscle Skeletal
Nerve Tibial
Skin Sun Exposed
Small Intestine Terminal Ileum
Spleen
Thyroid
Whole Blood
B
COBRA
Oxidative Phosphorylation
Electron Transport Chain Oxphos System In Mitochondria
The Citric Acid Tca Cycle And Respiratory Electron T...
Parkinsons Disease
Respiratory Electron Transport Atp Synthesis By Chem...
Translation
Complex I Biogenesis
Mitochondrial Translation
Metabolism Of Nucleotides
Translation Factors
Respiratory Electron Transport
Medicus Reference Electron Transfer In Complex I
Medicus Variant Mutation Caused Aberrant Snca To Ele...
Biological Oxidations
Phase Ii Conjugation Of Compounds
Metapathway Biotransformation Phase I And Ii
Chromatin Modifying Enzymes
2Q11 2 Copy Number Variation Syndrome
Phase I Functionalization Of Compounds
Snare Interactions In Vesicular Transport
Deregulation Of Rab And Rab Effector Genes In Bladde...
Peptide Hormone Metabolism
Digestion
Digestion And Absorption
Mitochondrial Complex I Assembly Model Oxphos System
Cellular Proteostasis
Histone Modifications
Genes
7 genes
22 genes
37 genes
-log10(padj)
0
5
10
15
20
25
30
35
40
Adipose Subcutaneous
Artery Tibial
Heart Left Ventricle
Lung
Muscle Skeletal
Nerve Tibial
Skin Sun Exposed
Small Intestine Terminal Ileum
Spleen
Thyroid
Whole Blood
