# Supplementary material for: Commentary on ‘corradjust unveils biologically relevant transcriptomic correlations by efficiently eliminating hidden confounders’
Source: Nucleic Acids Res. 2026 Oct 5;54(18):gkag932. doi: 10.1093/nar/gkag932 (PMC13635743; doi:10.1093/nar/gkag932)
Supplement: Supplementary file 1 [file gkag932_supplemental_files.zip › figS2.pdf]

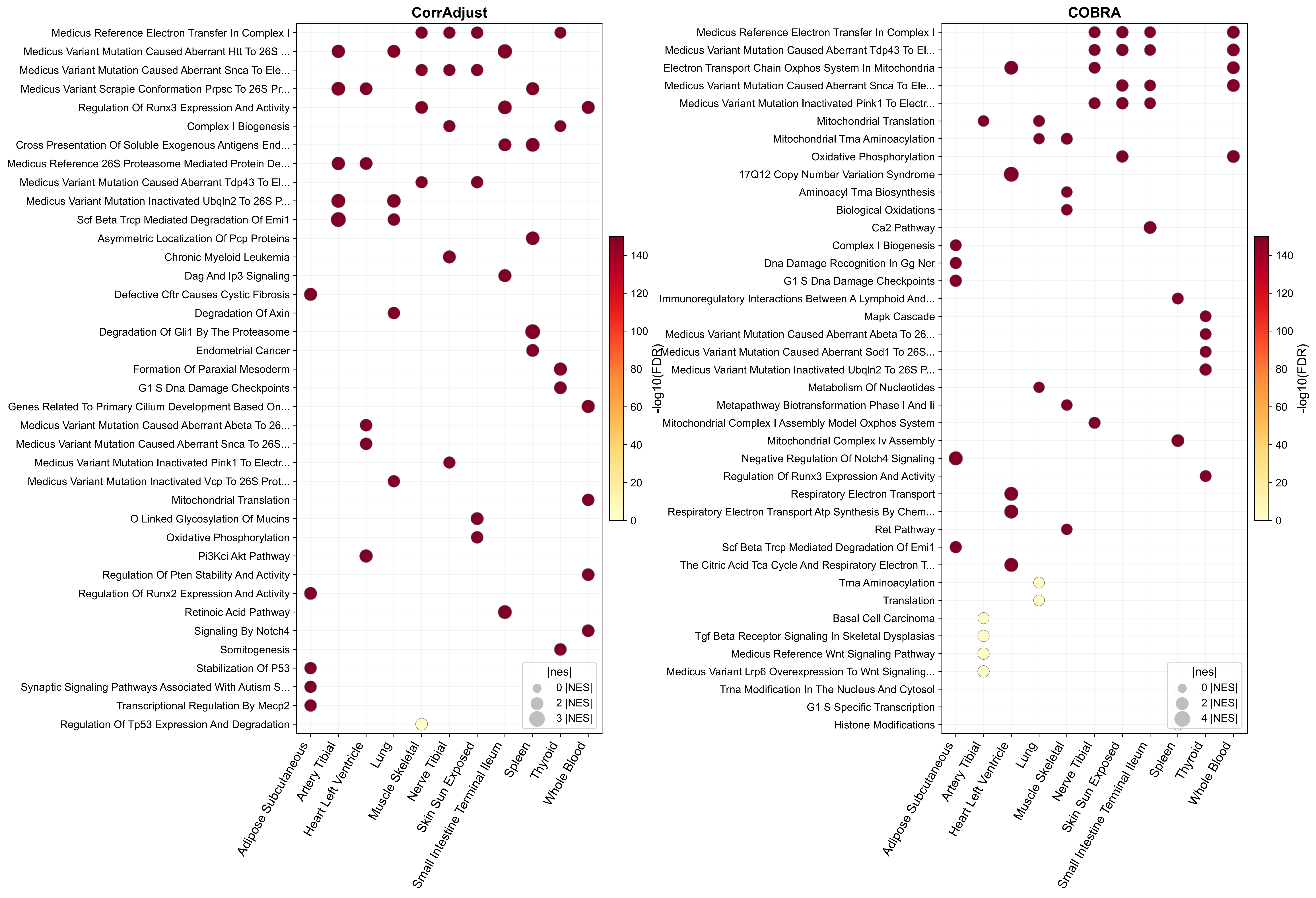

CorrAdjust
Medicus Reference Electron Transfer In Complex I
Medicus Variant Mutation Caused Aberrant Htt To 26S ...
Medicus Variant Mutation Caused Aberrant Snca To Ele...
Medicus Variant Scrapie Conformation Prpsc To 26S Pr...
Regulation Of Runx3 Expression And Activity
Complex I Biogenesis
Cross Presentation Of Soluble Exogenous Antigens End...
Medicus Reference 26S Proteasome Mediated Protein De...
Medicus Variant Mutation Caused Aberrant Tdp43 To El...
Medicus Variant Mutation Inactivated Ubqln2 To 26S P...
Scf Beta Trcp Mediated Degradation Of Emi1
Asymmetric Localization Of Pcp Proteins
Chronic Myeloid Leukemia
Dag And Ip3 Signaling
Defective Cftr Causes Cystic Fibrosis
Degradation Of Axin
Degradation Of Gli1 By The Proteasome
Endometrial Cancer
Formation Of Paraxial Mesoderm
G1 S Dna Damage Checkpoints
Genes Related To Primary Cilium Development Based On...
Medicus Variant Mutation Caused Aberrant Abeta To 26...
Medicus Variant Mutation Caused Aberrant Snca To 26S...
Medicus Variant Mutation Inactivated Pink1 To Electr...
Medicus Variant Mutation Inactivated Vcp To 26S Prot...
Mitochondrial Translation
O Linked Glycosylation Of Mucins
Oxidative Phosphorylation
Pi3Kci Akt Pathway
Regulation Of Pten Stability And Activity
Regulation Of Runx2 Expression And Activity
Retinoic Acid Pathway
Signaling By Notch4
Somitogenesis
Stabilization Of P53
Synaptic Signaling Pathways Associated With Autism S...
Transcriptional Regulation By Mecp2
Regulation Of Tp53 Expression And Degradation
Adipose Subcutaneous
Artery Tibial
Heart Left Ventricle
Lung
Muscle Skeletal
Nerve Tibial
Skin Sun Exposed
Small Intestine Terminal Ileum
Spleen
Thyroid
Whole Blood
|nes|
0 |NES|
2 |NES|
3 |NES|
-log10(FDR)
0
20
40
60
80
100
120
140
COBRA
Medicus Reference Electron Transfer In Complex I
Medicus Variant Mutation Caused Aberrant Tdp43 To El...
Electron Transport Chain Oxphos System In Mitochondria
Medicus Variant Mutation Caused Aberrant Snca To Ele...
Medicus Variant Mutation Inactivated Pink1 To Electr...
Mitochondrial Translation
Mitochondrial Trna Aminoacylation
Oxidative Phosphorylation
17Q12 Copy Number Variation Syndrome
Aminoacyl Trna Biosynthesis
Biological Oxidations
Ca2 Pathway
Complex I Biogenesis
Dna Damage Recognition In Gg Ner
G1 S Dna Damage Checkpoints
Immunoregulatory Interactions Between A Lymphoid And...
Mapk Cascade
Medicus Variant Mutation Caused Aberrant Abeta To 26...
Medicus Variant Mutation Caused Aberrant Sod1 To 26S...
Medicus Variant Mutation Inactivated Ubqln2 To 26S P...
Metabolism Of Nucleotides
Metapathway Biotransformation Phase I And Ii
Mitochondrial Complex I Assembly Model Oxphos System
Mitochondrial Complex Iv Assembly
Negative Regulation Of Notch4 Signaling
Regulation Of Runx3 Expression And Activity
Respiratory Electron Transport
Respiratory Electron Transport Atp Synthesis By Chem...
Ret Pathway
Scf Beta Trcp Mediated Degradation Of Emi1
The Citric Acid Tca Cycle And Respiratory Electron T...
Trna Aminoacylation
Translation
Basal Cell Carcinoma
Tgf Beta Receptor Signaling In Skeletal Dysplasias
Medicus Reference Wnt Signaling Pathway
Medicus Variant Lrp6 Overexpression To Wnt Signaling...
Trna Modification In The Nucleus And Cytosol
G1 S Specific Transcription
Histone Modifications
Adipose Subcutaneous
Artery Tibial
Heart Left Ventricle
Lung
Muscle Skeletal
Nerve Tibial
Skin Sun Exposed
Small Intestine Terminal Ileum
Spleen
Thyroid
Whole Blood
|nes|
0 |NES|
2 |NES|
4 |NES|
-log10(FDR)
0
20
40
60
80
100
120
140
