# Supplementary material for: Commentary on ‘corradjust unveils biologically relevant transcriptomic correlations by efficiently eliminating hidden confounders’
Source: Nucleic Acids Res. 2026 Oct 5;54(18):gkag932. doi: 10.1093/nar/gkag932 (PMC13635743; doi:10.1093/nar/gkag932)
Supplement: Supplementary file 1 [file gkag932_supplemental_files.zip › figS3.pdf]

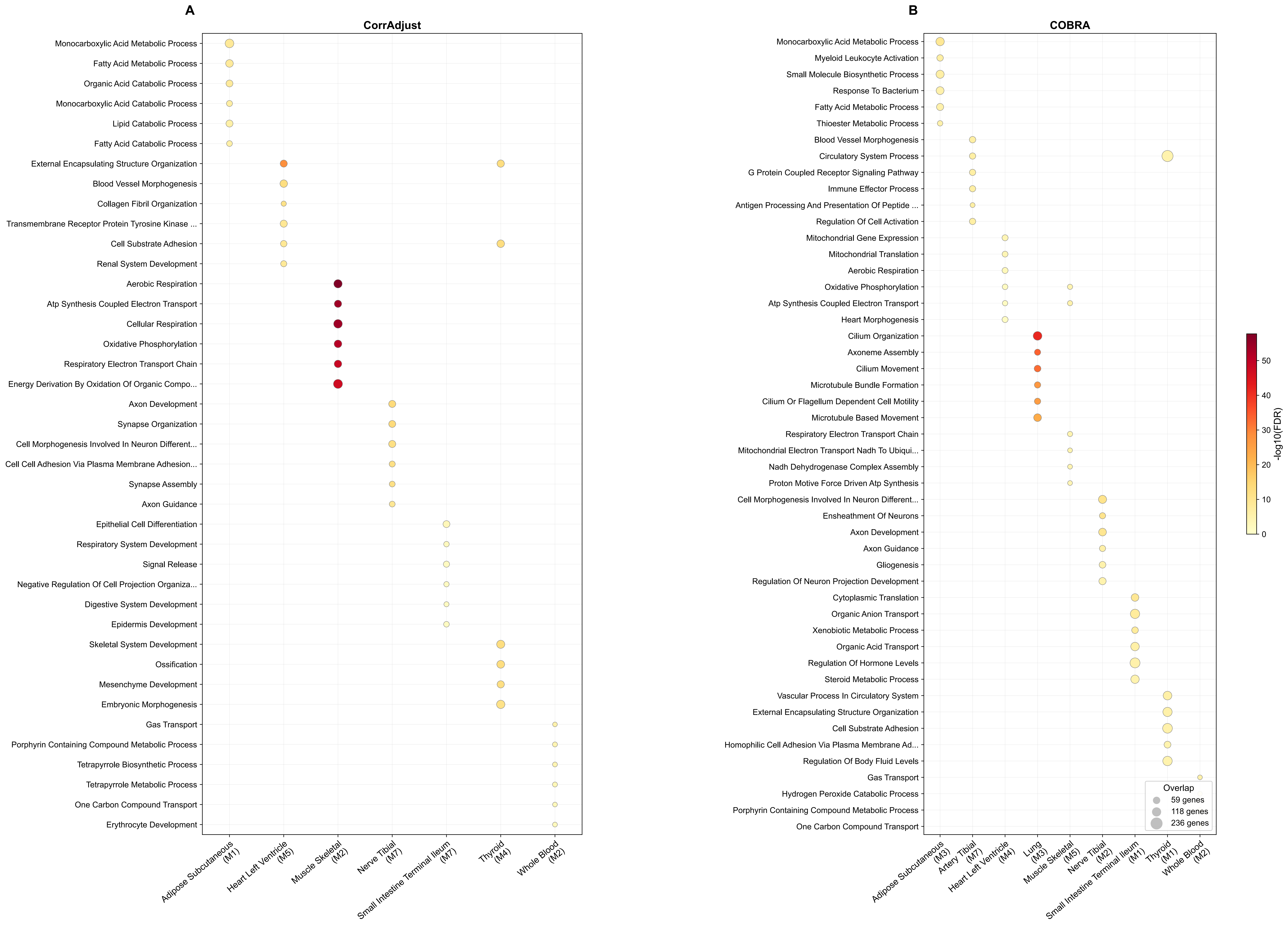
A
CorrAdjust
Monocarboxylic Acid Metabolic Process
Fatty Acid Metabolic Process
Organic Acid Catabolic Process
Monocarboxylic Acid Catabolic Process
Lipid Catabolic Process
Fatty Acid Catabolic Process
External Encapsulating Structure Organization
Blood Vessel Morphogenesis
Collagen Fibril Organization
Transmembrane Receptor Protein Tyrosine Kinase ...
Cell Substrate Adhesion
Renal System Development
Aerobic Respiration
Atp Synthesis Coupled Electron Transport
Cellular Respiration
Oxidative Phosphorylation
Respiratory Electron Transport Chain
Energy Derivation By Oxidation Of Organic Compo...
Axon Development
Synapse Organization
Cell Morphogenesis Involved In Neuron Different...
Cell Cell Adhesion Via Plasma Membrane Adhesion...
Synapse Assembly
Axon Guidance
Epithelial Cell Differentiation
Respiratory System Development
Signal Release
Negative Regulation Of Cell Projection Organiza...
Digestive System Development
Epidermis Development
Skeletal System Development
Ossification
Mesenchyme Development
Embryonic Morphogenesis
Gas Transport
Porphyrin Containing Compound Metabolic Process
Tetrapyrrole Biosynthetic Process
Tetrapyrrole Metabolic Process
One Carbon Compound Transport
Erythrocyte Development
Adipose Subcutaneous (M1)
Heart Left Ventricle (M5)
Muscle Skeletal (M2)
Nerve Tibial (M7)
Small Intestine Terminal Ileum (M7)
Thyroid (M4)
Whole Blood (M2)
B
COBRA
Monocarboxylic Acid Metabolic Process
Myeloid Leukocyte Activation
Small Molecule Biosynthetic Process
Response To Bacterium
Fatty Acid Metabolic Process
Thioester Metabolic Process
Blood Vessel Morphogenesis
Circulatory System Process
G Protein Coupled Receptor Signaling Pathway
Immune Effector Process
Antigen Processing And Presentation Of Peptide ...
Regulation Of Cell Activation
Mitochondrial Gene Expression
Mitochondrial Translation
Aerobic Respiration
Oxidative Phosphorylation
Atp Synthesis Coupled Electron Transport
Heart Morphogenesis
Cilium Organization
Axoneme Assembly
Cilium Movement
Microtubule Bundle Formation
Cilium Or Flagellum Dependent Cell Motility
Microtubule Based Movement
Respiratory Electron Transport Chain
Mitochondrial Electron Transport Nadh To Ubiqui...
Nadh Dehydrogenase Complex Assembly
Proton Motive Force Driven Atp Synthesis
Cell Morphogenesis Involved In Neuron Different...
Ensheathment Of Neurons
Axon Development
Axon Guidance
Gliogenesis
Regulation Of Neuron Projection Development
Cytoplasmic Translation
Organic Anion Transport
Xenobiotic Metabolic Process
Organic Acid Transport
Regulation Of Hormone Levels
Steroid Metabolic Process
Vascular Process In Circulatory System
External Encapsulating Structure Organization
Cell Substrate Adhesion
Homophilic Cell Adhesion Via Plasma Membrane Ad...
Regulation Of Body Fluid Levels
Gas Transport
Hydrogen Peroxide Catabolic Process
Porphyrin Containing Compound Metabolic Process
One Carbon Compound Transport
Overlap
59 genes
118 genes
236 genes
Adipose Subcutaneous (M3)
Artery Tibial (M7)
Heart Left Ventricle (M4)
Lung (M3)
Muscle Skeletal (M5)
Nerve Tibial (M2)
Small Intestine Terminal Ileum (M1)
Thyroid (M1)
Whole Blood (M2)
-log10(FDR)
0
10
20
30
40
50
