# Supplementary material for: Commentary on ‘corradjust unveils biologically relevant transcriptomic correlations by efficiently eliminating hidden confounders’
Source: Nucleic Acids Res. 2026 Oct 5;54(18):gkag932. doi: 10.1093/nar/gkag932 (PMC13635743; doi:10.1093/nar/gkag932)
Supplement: Supplementary file 1 [file gkag932_supplemental_files.zip › Supplementary Data.docx]

**Supplementary material**

**COBRA regression modes**

COBRA decomposes a gene co-expression matrix into distinct submatrices for each covariate. To do so “pseudo-eigenvalues” of these submatrices are derived by determining the contribution of each covariate towards the eigenvalues of the co-expression matrix, which comes in three variants.

1- Maximum Likelihood Estimator (MLE)

$${argmin}_{\psi}\frac{1}{2}\left\| T-X\psi^{T} \right\|_{F}^{2}$$

With $T$ the matrix of contribution of sample $i$ to eigenvector $j$, i.e., $T_{j,i}=Q_{.,j}^{T}G_{.,i}G_{.,i}^{T}Q_{.,j}$, $\psi$ is the matrix of pseudo-eigenvalues: $\psi_{k,j}$ is the contribution of covariate $k$ to eigenvalue $j$ of the co-expression matrix, and $X$ the design matrix. Although matrices produced are biologically meaningful, we also provide options to derive valid positive semi-definite matrices by constraining positive eigenvalues.

2- Nonnegative Least Squares (NNLS)

$${argmin}_{\psi\geq0}\frac{1}{2}\left\| T-X\psi^{T} \right\|_{F}^{2}$$

Using similar notations.

3- Nonnegative Lasso (NNLASSO)

$${argmin}_{\psi\geq0}\frac{1}{2}\left\| T-X\psi^{T} \right\|_{F}^{2}+{\lambda\left\| \psi\right\|}_{1}$$

With $\lambda$ the l1 regularization parameter.

**Supplementary figures**

Figure S1 - Over-representation analysis of the top 100 genes. Genes were ranked by enriched score as defined in CorrAdjust. Enriched terms in (A) CorrAdjust and (B) COBRA networks across 11 GTEx tissues in the Canonical Pathways database. Enriched pathways are not tissue specific.

Figure S2 - GSEA of genes in 11 GTEx tissue networks. Genes were ranked by enrichment score as defined in CorrAdjust. Pathway enrichment analysis of (A) CorrAdjust and (B) COBRA networks in the Canonical Pathways database. This analysis shows that enriched pathways are not tissue specific.

Figure S3 - Tissue-specific modules across 11 GTEx tissues in (A) CorrAdjust and (B) COBRA networks. Here, we identified modules then did over-representation analysis of all the genes in each module. For each tissue, we show the module that represents tissue-specific functions. These modules were selected manually. For example, in adipose tissue networks, module 1 in CorrAdjust and module 3 in COBRA represent tissue-specific functions, e.g., fatty acid metabolic process.
